# Supplementary material for: Anthocyanin-Rich Extract Mitigates the Contribution of the Pathobiont Genus Haemophilus in Mild-to-Moderate Ulcerative Colitis Patients
Source: Microorganisms. 2024 Nov 20;12(11):2376. doi: 10.3390/microorganisms12112376 (PMC11596099; doi:10.3390/microorganisms12112376)
Supplement: Supplementary file 1 [file microorganisms-12-02376-s001.zip › microorganisms-3274189-supplementary.pdf]

microorganisms

Supplementary Table 1: Detailled inclusion and exclusion criteria

Inclusion Criteria	Exclusion Criteria
Male or female ≥18 years of age	Acute fulminant UC and/or signs of systemic toxicity
Established diagnosis of UC, with minimum time from diagnosis of ≥3 months	UC limited to the rectum (disease which extends <15cm above the anal verge)
Moderately at least left sided UC (disease should extend 15cm or more above the anal verge). Disease severity determined by a Modified Mayo Score of 6 to 12 with an endoscopic sub score ≥2 assessed by central reading of endoscopy performed at screening visit and no other individual sub score <1	Suspicion of differential diagnosis such as: Crohn's, enterocolitis, ischaemic colitis, radiation colitis, indeterminate colitis, infectious colitis, diverticular disease, associated colitis, microscopic colitis, massive pseudopolyposis or non-passable stenosis
Current oral or rectal 5-ASA/SP use or a history of oral or rectal 5-ASA/SP use	Serious active infection or current history of active infection<br>Gastrointestinal infections including positive Clostridium difficile stool assay
Current steroids use or history of steroids dependency, refractory, or intolerance, including no steroids treatment due to earlier side-effects (only one of the steroids criteria have to be fulfilled, see definition in European Crohn's and Colitis organization (ECCO) guidelines)	History or presence of any clinically significant disorder that, in option of the investigator, could impact on patient's possibility to adhere to the protocol and protocol procedures or would confound the study result or compromise patient safety
One of the following points must be fulfilled:<br>• Active disease despite induction therapy with 5-ASA agents where adequate therapy is considered with an oral 5-ASA (mesalamine 2-4.8g/day, sulfasalazine 4-6g/day) administered for at least 2 weeks. Topical treatment with 5-ASA may have been attempted but this is not a prerequisite for inclusion in the study<br>• Intolerance to oral 5-ASAs or azathioprine<br>• Active disease despite a thiopurine (adequately dosed according to treatment guidelines, such as 2-3 mg/kg for azathioprine) or methotrexate administered for at least 12 weeks<br>• Active disease despite treatment with biologicals or calcineurin inhibitors	History of malignancy, except for:<br>• Treated (cured) basal cell or squamous cell in situ carcinoma<br>• Treated (cured) cervical intraepithelial neoplasia<br>• Carcinoma in situ of the cervix with no evidence of recurrence within the previous 5 years prior to the screening visit<br>Presence or history of underlying metabolic, endocrine, hematologic, pulmonary, cardiac, blood, renal, hepatic, infectious, psychiatric or any medically unstable condition as assessed by the primary treating physician which, in the option of the investigator, would place the subject at unacceptable risk for participation in the study<br>Significant illness within the two weeks prior to dosing or any active infection or medical condition that may require treatment or therapeutic intervention during the study<br>Known history of alcohol abuse, chronic liver or biliary disease<br>History or presence of a significant renal disease
Allowed to receive a therapeutic dose of following UC drugs during the study:<br>• Oral steroids therapy (≤30mg prednisone or equivalent/day) providing that the dose has been stable for 2 weeks prior to baseline<br>• Oral or rectal MMX Budesonide therapy (9mg/day) initiated at least 8 weeks before baseline<br>• Oral or rectal 5-ASA/SP compounds, providing that the dose has been stable for 2 weeks before baseline visit<br>• AZA/6-MP providing that the dose has been stable for 8 weeks prior to baseline and been initiated at least 2 months before screening<br>• TNF inhibitors (Infliximab, Adalimumab or Golimumab) are allowed, providing that the dose is stable for at least 2 months prior to baseline and during the study treatment period<br>• Vedolizumab and Tofacitinib is allowed, providing that the dose is stable for at least 2 months prior to baseline and during the study treatment period	Long term treatment with antibiotics or non-steroidal anti-inflammatory drugs (NSAIDs) within two weeks prior to screening (one short treatment regime for antibiotics and occasional use of NSAIDs are allowed)<br>Antibiotics within the last week prior to randomization, subjects who received cyclosporine or tacrolimus within the last 4 weeks prior to randomization and a dose of >30mg/day or equivalent per kg body weight in the last 4 weeks prior to randomization<br>Subjects who have been treated with a dose of ≥1mg per kg body weight prednisone or ≥30mg/day in the last 4 weeks prior to randomization<br>Ongoing treatment with cyclosporine or tacrolimus. Eligible subjects must have stopped cyclosporine and tacrolimus at least 4 weeks and antibiotics at least 1 week prior to randomization.<br>Currently receiving parenteral nutrition or blood transfusions<br>Planned diet change, any severe or new dietary restrictions<br>Known allergies to bilberries or any other AC containing fruits
Ability to understand the treatment, willingness to comply all study requirements and ability to provide informed consent	Concurrent participation in another clinical study with investigational therapy or previous use of investigational therapy within 5 half-lives and within at least 30 days after last treatment of the experimental product prior to enrolment<br>Females who are lactating or have a positive serum pregnancy test during the screening period<br>Repeated and confirmed laboratory findings showing<br>• total bilirubin >2xULN unless in Gilbert's syndrome<br>• AP >2xULN<br>• ALT>2xULN<br>• serum creatinine >2xULN<br>• white blood cell count outside the range of 3,000-15,000 per µL<br>• platelet count < 100,000 per µl<br>• hemoglobin <8g per dl or other signs of severe anemia

Supplementary Table 2: Detailed study procedure description

**Study Periods**	**Screening**	**Treatment Period**				**Follow-up**
**Visit**	**S**	**B**	**1**	**2**	**3**	**FUP**
**Day**	**-28 to -0**	**1 = Baseline**	**15± 4d**	**28± 4d**	**56± 4d**	**86± 4d**
Subject Information and Informed Consent	x					
Demographics	x					
Medical History	x					
Update Medical History		x	x	x	x	x
In-/Exclusion Criteria	x	x				
Physical Examination	x	x	x	x	x	x
Vital Signs (pulse, blood pressure, temperature), Body Weight, Body Height[1]	x	x	x	x	x	x
Serum Laboratory Tests (Safety Lab) 2	x	x	x	x	x	x
Pregnancy Test[3]	x	x	x	x	x	x
Urine analysis	x	x	x	x	x	x
Fecal Samples, stool bacteriology, C. diff.	x					
Fecal Calprotectin	x	x	x	x	x	x
Microbiota Analyses	x	x	x	x	x	x
Resting-ECG	x					
Sigmoidoscopy with 6 Biopsies and Endoscopic Mayo Subcore and Histology (Geboes Index) Recording for central reading	x				x	
Complete Mayo Score	x				x	
Partial Mayo Score (including PRO2)	x	x	x	x	x	x
Dispense Subject Diaries [4]	x	x	x	x	x	
Collect Subject Diaries[4]		x	x	x	x	x
Assess quality of life (SIBDQ[4], EuroQoL5D)	x	x	x	x	x	x
Distribution and/ or collecting of Study Medication		x	x	x	x	x (if applicable)
Concomitant Therapy	x	x	x	x	x	x
Adverse Events and Serious Adverse Events		x	x	x	x	x
[1] only at screening<br>[2] including hematology, blood chemistry<br>[3] Screening with serum pregnancy test, other visits with urine stick test<br>[4] SIBDQ = Short Inflammatory Bowel Disease Questionnaire, Patient Diary (including stool frequency, abdominal pain and general well-being) indicated in appendix						

Supplementary Table 3: Results of correlation and their p-value calculations of the most strongly (negatively) with calprotectin

Genus	n	estimate	conf.low	conf.high	p.value
Haemophilus	53	0.2593757	0.1137147	0.4050367	0.0008317
Parasutterella	46	-0.2207625	-0.3452672	-0.0962579	0.0009309
TF01-11	44	0.1924920	0.0588096	0.3261744	0.0057802
Hungatella_A	42	0.2343540	0.0531916	0.4155164	0.0122185
Proteus	13	0.3050273	0.0675042	0.5425505	0.0127660
Lachnospira	64	0.2936038	0.0633476	0.5238600	0.0133537
Campylobacter_B	16	0.6225038	0.1308248	1.1141828	0.0140027
Limosilactobacillus	17	0.3172125	0.0462716	0.5881534	0.0226064
Lawsonibacter	65	-0.3171477	-0.6015451	-0.0327502	0.0295512
Phascolarctobacterium	34	-0.1352149	-0.2594201	-0.0110097	0.0338408
An114	54	-0.2311532	-0.4504749	-0.0118315	0.0392678
Neobitarella	14	0.5068346	0.0161696	0.9974996	0.0431712
Anaerobutyricum	62	-0.2389307	-0.4762729	-0.0015885	0.0485682

Supplementary Figure 1a, b & c: Change in fecal calprotectin throughout the study

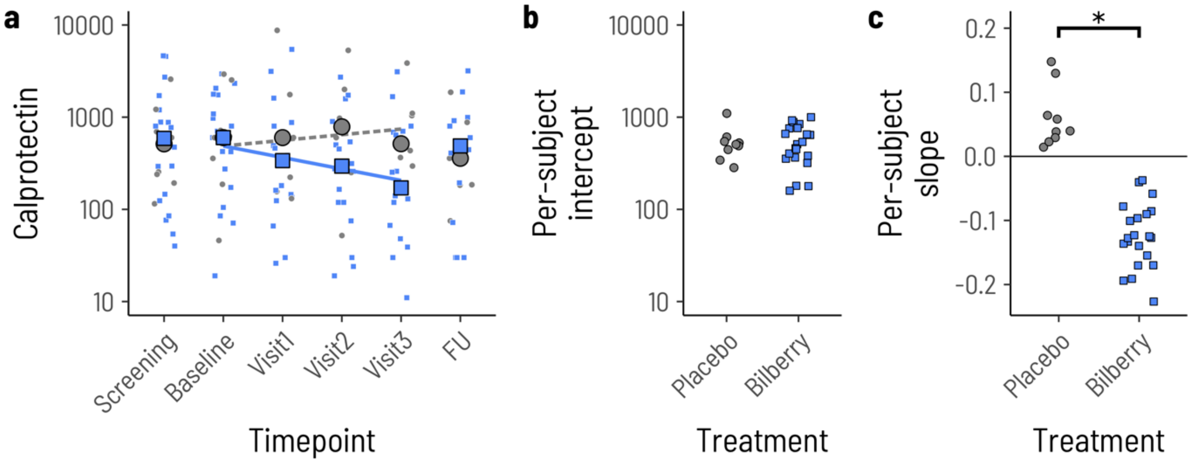

__a.__ Each point shows the concentration of fecal calprotectin (y-axis) in placebo-treated (grey) or bilberry-treated (blue) subjects. The large circles and squares show the group means. The dashed and solid lines the mean slope for placebo and bilberry, respectively, estimated from a linear mixed model with random slopes and intercepts for the two groups. __b.__ Estimated intercept for

Supplementary Figure 2: The positive correlation of Haemophilus and Calprotectin remains while placebo intake

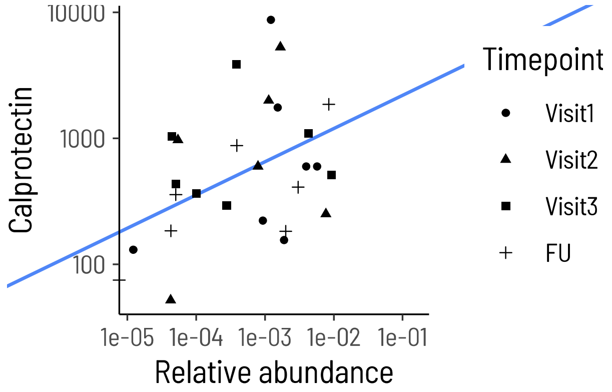

Visualization of relationship between relative abundance (x-axis) and calprotectin (y-axis) of Haemophilus during the intervention in the placebo group. A single dot
